# NICU nurses’ experiences of providing internet-based nursing consultation services: a descriptive qualitative study

**DOI:** 10.3389/fped.2026.1950395

**Published:** 2026-10-08

**Authors:** Jiahua Zhang, Yajing Wang, Yao Chen, Yuting Xie, Shuju Feng

**Affiliations:** Neonatal Intensive Care Unit, Peking Union Medical College Hospital, Chinese Academy of Medical Sciences & Peking Union Medical College, Beijing, China

**Keywords:** experience, internet plus healthcare, internet-based nursing consultation, NICU, qualitative study

## Abstract

**Objective:**

To explore the experiences, perceived barriers, risk concerns, and support needs of neonatal intensive care unit (NICU) nurses providing internet-based nursing consultation services to parents and other caregivers of newborns.

**Methods:**

A descriptive qualitative study was conducted in the NICU of a tertiary hospital in Beijing, China. Purposive sampling was used to recruit nurses with direct experience in providing internet-based nursing consultations. Twelve NICU nurses participated in individual, face-to-face, semi-structured interviews conducted between April and May 2026. Interviews were audio-recorded, transcribed verbatim, and returned to participants for participant transcript verification. Data were analyzed using inductive content analysis based on the approach of Graneheim and Lundman. Two researchers independently conducted coding and reached consensus through discussion. The study was reported in accordance with the Consolidated Criteria for Reporting Qualitative Research (COREQ).

**Results:**

Four themes and nine subthemes emerged from the data. Theme 1, perceived value of internet-based nursing consultation, included highlight clinical service value and enhance personal professional value. Theme 2, barriers to implementation of internet-based nursing consultation, comprised imperfect platform functionality, inadequate consultation matching and information acquisition, and lack of institutional development and insufficient platform promotion. Theme 3, heightened risk perception and responsibility pressure, included clinical judgment risks, and communication and reputational risks. Theme 4, negotiating work demands and resource support, comprised flexible working hours and workload balance and resource support and training needs. Participants perceived internet-based nursing consultation as a convenient way to extend professional nursing support and enhance nurses' sense of professional value. However, inadequate platform functionality, inappropriate consultation matching, incomplete information, clinical uncertainty, communication risks, workload pressures, and insufficient organizational support constrained service delivery.

**Conclusion:**

Internet-based nursing consultation may extend specialized nursing support to families of newborns and provide nurses with opportunities for professional development and value affirmation. However, participants' accounts suggest that sustainable implementation may benefit from clear boundaries of nursing consultation, appropriate referral and escalation pathways, improved consultation matching and platform functionality, workload and specialist-resource support, and targeted training for managing complex and uncertain neonatal care concerns.

## Introduction

## 1 Background

In recent years, the rapid advancement of internet healthcare has become a global trend, transforming traditional healthcare delivery models and promoting the integration of digital technologies into health services worldwide (1). In China, the government issued guidelines to encourage the development of “Internet Plus Healthcare” in 2018, accelerating the establishment of internet hospitals and the expansion of online healthcare services (2). Subsequently, the “Internet Plus Nursing Service” model was introduced as an important extension of internet healthcare, aiming to integrate nursing services with digital platforms and improve accessibility to professional nursing support (3). Within this framework, internet-based nursing consultation services have emerged. This service model enables qualified nurses to provide nursing consultation, health education, and professional nursing guidance through internet hospital healthcare platforms. Patients can access the platform at any time to select an appropriate nurse and make an appointment according to their needs (4). Internet-based nursing consultation services belong to an expanding field of practice, and there is currently no standardized operational or practice guideline available. Specific operational models vary across healthcare institutions due to their individual circumstances, the overall service objectives, basic framework, and general workflow remain largely consistent.

Traditional continuity of care is often organized around the transition of patients from hospital-based care to subsequent care settings, requiring coordination, information exchange, and follow-up between healthcare professionals across these settings (5). In contrast, internet-based nursing consultation services expand the accessibility and reach of nursing support by enabling patients and families to obtain professional nursing guidance remotely, regardless of their geographical location or whether they are currently receiving care within a healthcare institution. Patients and their families can communicate with nurses via text, voice, images, or video to receive timely nursing support (4). Internet-based nursing consultation has been increasingly applied in China across a range of patient populations, including individuals with enterostomies, breastfeeding problems and neonatal conditions (6–8).These studies have demonstrated that this model can enhance disease management, improve patients' self-care abilities and quality of life, and provide more convenient and timely health guidance for parents and other caregivers of newborns, while also alleviating anxiety and generating potential social benefits.

However, existing studies have predominantly focused on evaluating the effectiveness and outcomes of internet-based nursing consultation from the perspective of patients and families, whereas relatively little attention has been paid to the experiences of healthcare professionals involved in delivering such services. A qualitative study by Zhao et al. explored nurses' perceptions of participating in internet-based nursing services and identified concerns related to professional competencies, organizational support, family responsibilities, and patient and nurse safety (9). However, the services examined in that study primarily involved home-based nursing procedures, such as injections, blood collection, rather than internet-based nursing consultation and health guidance alone. Therefore, its findings may not fully capture the experiences and challenges associated with providing nursing consultation remotely without direct physical contact with patients. Studies have also explored healthcare professionals' experiences with internet-based consultation from the perspective of physicians (10); however, the experiences of nurses who provide internet-based nursing consultation remain insufficiently understood.

This gap may be particularly important in neonatal care. Newborns and their families may seek internet-based nursing consultation for a wide range of health-related concerns, including feeding difficulties, jaundice, sleep, growth and development, infection prevention, medication administration, and the recognition of potentially concerning symptoms (11). Such consultations may involve not only infants discharged from the NICU but also newborns who have never been hospitalized but whose parents and other caregivers require professional guidance during early infancy. For NICU nurses, providing such consultation may be particularly challenging because newborns are unable to communicate their symptoms, while nurses must rely largely on parents and other caregivers' descriptions and, when available, photographs or videos rather than direct observation and physical assessment. The complexity of neonatal conditions, the vulnerability of this population, and the need to distinguish routine caregiving concerns from potentially serious health problems may therefore place unique demands on nurses' clinical judgment, communication, professional responsibility, and risk management. Therefore, this study aimed to explore NICU nurses' experiences, perceived barriers, risk concerns, and support needs when providing internet-based nursing consultation services, which may provide important insights into the challenges and support needs associated with delivering specialized nursing guidance to newborns and their families through digital platforms.

## 2 Method

### 2.1 Study design and setting

A descriptive qualitative design was adopted to obtain a comprehensive and straightforward description of NICU nurses' experiences, perceived barriers, risk concerns, and support needs when providing internet-based nursing consultation services. We recruited nurses from NICU of a Grade A tertiary hospital in Beijing, China. This hospital was among the first to pilot internet-based nursing services.

### 2.2 Internet-based nursing consultation service context

The internet-based nursing consultation service was established within the hospital's internet healthcare platform to provide professional neonatal nursing consultation and health guidance for parents and other caregivers of newborns. The service was not limited to infants previously discharged from the hospital's NICU and was available to families seeking professional support for neonatal care.

Nurses participating in the service were required to meet the hospital's eligibility criteria, which included being a registered nurse holding a valid Nurse Practicing Certificate of the People's Republic of China, demonstrating good professional ethics, having at least 5 years of clinical nursing experience, and holding a professional title of senior nurse or above. Nurses with specialized nursing qualifications or relevant specialty nursing experience were preferred. To further ensure the quality of neonatal nursing consultations, the NICU adopted a stricter criterion, requiring participating nurses to have at least 10 years of clinical nursing experience and hold specialized nurse qualifications. Nurses who met the criteria were eligible to receive standardized training provided by the hospital on internet-based nursing consultation.

The service operated through an appointment-based model that combined asynchronous appointment and information submission with real-time communication after a consultation was accepted. It did not have fixed operating hours that applied uniformly to all participating nurses. Instead, nurses independently determined the dates on which they were available for consultation and the number of appointment slots they would offer each day. Thus, although parents and other caregivers could make appointments through the platform at any time, the actual availability of consultation depended on the consultation slots opened by individual nurses. At the time of booking, parents and other caregivers could upload relevant medical information and briefly describe their concerns using text and photographs. Once a nurse accepted the consultation, further communication could take place through text messages, photographs, voice calls, or video calls, according to the needs of the consultation. Consultation requests were not actively assigned to nurses by the platform. Instead, nurses were presented to parents and other caregivers in an alphabetical order based on the first letter of the Pinyin spelling of the surname, and parents and other caregivers selected a nurse according to their perceived consultation needs.

Nurses could provide consultations during both working and non-working hours. Because nurses continued to undertake their routine clinical responsibilities and might also provide consultations during personal time, a standardized response time could not be guaranteed. The number of consultations depended on the number of consultation days that NICU nurses arranged for themselves and the available slots per day. The duration of a consultation depended on the complexity of the needs and ends when the issue was ultimately resolved. The service was provided free of charge throughout the study period.

At the time of the study, the platform also lacked an automated triage function that could systematically match consultation requests with nurses according to their areas of expertise. When a consultation involved issues beyond the nurse's scope of practice, nurses did not provide diagnostic or treatment advice or directly transfer the consultation to a physician. Instead, they advised parents and other caregivers to seek care from an appropriate medical specialty through an online or in-person consultation, which parents and other caregivers arranged independently. Consultation records were retained within the internet-based nursing consultation platform but were not incorporated into the patients' formal electronic health records.

### 2.3 Recruitment and sampling

Purposive sampling was used to recruit nurses with direct experience in internet-based nursing consultation. The inclusion criteria were: (1) registered nurses with a professional title of senior nurse or above; (2) at least 10 years of NICU clinical experience; (3) specialist nurse qualifications; and (4) direct experience providing internet-based nursing consultation in the study setting. The exclusion criterion was: nurses who were unable to complete the interview because of prolonged leave, external training, or other circumstances that prevented participation during the study period.

In the study setting, 16 nurses were initially screened and identified as potentially eligible based on the institutional requirements for providing internet-based nursing consultation. During the screening process, four nurses were excluded: two had not registered for the internet-based nursing consultation service, and two were unavailable for participation at the time of recruitment (one was on maternity leave and one was away for training). The remaining 12 nurses met all final eligibility criteria and were invited to participate in the study. All 12 invited nurses agreed to participate, resulting in a final sample of 12 participants. One of the researchers, a nurse manager (SJ. F) informed the principal researcher (JH. Z) of nurses who met the eligibility criteria, but because SJ. F had authority over participants' employment, performance evaluation, scheduling, promotion, or access to consultation work, she did not participate in recruitment or approach potential participants. The principal researcher independently approached the eligible nurses, explained the study, and invited them to participate.

### 2.4 Data collection

Following a literature review and consultations with neonatal care specialists and nursing administrators, a preliminary interview outline was developed. A pilot interview was conducted with SJ. F, and the final interview outline was developed after optimization. The pilot interview was not included in the final dataset or analysis. The interviews focused on three key areas:

(1) overall understanding of internet-based nursing consultations; (2) practical experiences with internet-based nursing consultations; (3) factors promoting and hindering the practice of internet-based nursing consultations. The complete interview outline is presented in Table 1.

**Table 1:** Outline of the interview.

Number	Questions
1	How did you initially learn about the internet-based nursing consultation service model?
2	What is your overall view of this service model?
3	What are your thoughts on applying this model to the field of neonatal care?
4	What were your feelings when you first encountered and actively participated in this model?
5	Could you share your personal experiences in practicing internet-based nursing consultations?
6	Based on your experience, what potential advantages and challenges do you see in internet-based nursing consultations?

The researcher (JH. Z) conducted 12 in-depth face-to-face interviews in a quiet departmental conference room between April 2026 and May 2026. Each interview lasted between 35 and 55 min. Before each interview, participants were informed of the identity and the professional background of the interviewer, the study's purpose and assured of privacy and confidentiality. Recordings were made by audio after obtaining consent. During the interviews, nonverbal cues such as body language and facial expressions were observed, and clarifying or follow-up questions were posed as needed.

Within 24 h after each interview, two researchers (JH. Z & YJ. W) independently transcribed each interview recording into text data. Any discrepancies were resolved by referring to the original audio recordings and through discussion between the two researchers. After reaching consensus on the transcripts, the finalized transcripts were returned individually to all participants (*N* = 12) for participant transcript verification. Participants were asked to review their transcripts and were invited to identify any inaccuracies, omissions, or statements they wished to modify or delete. All participants who reviewed the transcripts confirmed the accuracy of the content, and no participant requested substantive corrections or deletion of interview material. The data were then imported into NVivo 11.0 software for analysis and management.

All interviews were conducted in Mandarin Chinese, and the transcripts were transcribed in the same language. All stages of data analysis, including the identification and condensation of meaning units, coding, and the development and refinement of subthemes and themes, were conducted in Mandarin Chinese. After the analysis and coding framework had been finalized, the materials selected for presentation in the manuscript and Supplementary Table S1, including participant quotations, meaning units, condensed meaning units, codes, subthemes, and themes, were translated into English by YT. X and YJ. W, who were native Mandarin speakers, fluent in English, with experience in nursing-related training in an English-speaking country. The translations were checked against the original Mandarin transcripts by JH. Z who has a background in nursing and experience in academic writing in both Chinese and English. Differences were discussed and resolved through bilingual consensus, with particular attention to preserving the conceptual meaning, contextual meaning, and culturally specific expressions of participants' accounts.

### 2.5 Data analysis

Interview data were analyzed using inductive content analysis following the approach described by Ulla H Graneheim and Berit Lundman (12). The analysis focused primarily on the manifest content of the interviews, while the contextual meaning of participants' accounts was considered throughout the analytical process. The unit of analysis was a meaning unit, defined as a word, phrase, sentence, or group of sentences containing information relevant to the research questions. The researchers (JH. Z & YJ. W) read and reread the interview transcripts to become familiar with the data. Meaning units relevant to the study objectives were identified and condensed while retaining their essential meaning. All the condensed meaning units were then assigned descriptive codes by the two researchers independently. Differences in coding were discussed between the two researchers until consensus was reached. A third researcher was not required to resolve disagreements. Then, codes with similar meanings were compared, grouped, and organized into subthemes. Related subthemes were subsequently organized into overarching themes. The coding framework was developed iteratively throughout the analysis (Supplementary Table S1 coding framework). When new codes or concepts emerged that could not be adequately accommodated within the existing framework, the researchers revisited previously coded transcripts and modified the framework accordingly.

The research team continuously compared codes, themes with the original transcripts to ensure that the emerging findings remained grounded in the participants' accounts. The final themes were reviewed against the entire dataset, including examination of deviant or contradictory accounts, to determine whether they adequately captured the range and variation of participants' experiences. Data analysis proceeded iteratively, and the analysis was considered complete when the research team judged that the dataset had been sufficiently represented by the final coding structure and that no substantively new theme relevant to the research questions were emerging.

Because all 12 eligible service-providing nurses were interviewed, sample size was determined primarily by the eligible participant pool. Following completion of the interviews, the research team assessed thematic sufficiency and found that later interviews did not generate substantively new themes. Following team discussion, the researchers agreed that the existing thematic structure adequately captured the major content relevant to the research questions. Thematic saturation was therefore considered to have been achieved. No further interviews were conducted thereafter. The saturation assessment was documented through the final thematic framework and records of the research team's discussions.

The reporting of the qualitative study was guided by the Consolidated Criteria for Reporting Qualitative Research (COREQ). Details presented in Supplementary Table S3—COREQ Checklist.

### 2.6 Ethical considerations

This study has been approved by the Ethics Review Committee of Peking Union Medical College Hospital, Chinese Academy of Medical Sciences (I-23PJ232). All participants received a comprehensive explanation of the research and signed written informed consent forms. Interviews were anonymized through coding to ensure data confidentiality.

### 2.7 Trustworthiness and reflexivity

Trustworthiness was addressed through credibility, dependability, confirmability, and transferability. Credibility was enhanced by recruiting nurses with direct experience in internet-based nursing consultations, using open-ended and non-judgmental questions, and conducting researcher triangulation during coding and interpretation. Interviews were conducted privately, and participants were encouraged to express both positive and negative experiences.

Dependability and confirmability were supported by maintaining an audit trail documenting data collection, coding, theme development, and analytical decisions, with discrepancies discussed among the research team. Transferability was supported by providing detailed descriptions of the study setting, participants, eligibility criteria, and consultation model, enabling to assess the applicability of the findings to similar contexts. In addition, the interviewer (JH. Z), a female，held a master degree and was a registered nurse with the professional title of nurse. She had participated in the Eighth Theoretical and Practical Workshop on Qualitative Research at the School of Nursing, Fudan University, and had relevant experience in qualitative research. Although she worked in the same NICU as the participants, she had joined the department less than 6 months before the study and was not a supervisor or manager. She had no authority over participants' employment, performance evaluation, scheduling, promotion, or access to consultation work. To minimize potential social-desirability and hierarchical-response bias, participation was voluntary, and participants were informed that their participation and responses would not affect their employment, performance evaluation, work scheduling, promotion, or professional opportunities.

## 3 Results

### 3.1 Study participants

A total of 12 interviewees were finally included. All participants were from a large Grade A Tertiary hospital in Beijing, China. The participants were all female, had a mean age of (38.33 ± 5.14) years, the average number of years of participation in internet-based nursing consultation was (4.33 ± 2.06) years, and the average work experience in NICU was (14.58 ± 4.60) years. All participants are specialized nurses. Characteristics of the participants are shown in Table 2.

**Table 2:** Demographic data of the participants.

Characteristic	*n* (%)/Mean ± SD(Range)
Age, years	38.33 ± 5.14 (31–49)
Educational level	
Bachelor's degree	10 (83.33%)
Master's degree in Nursing	2 (16.67%)
NICU nursing experience, years	14.58 ± 4.60 (10–28)
Specialized Nurse Certification	
Wound and ostomy specialist	1 (8.33%)
Neonatal specialist nurse	9 (75.00%)
Intravenous therapy specialist nurse	2 (16.67%)
Formal nursing position^a^	
With formal position	6 (50.00%)
Without formal position	6 (50.00%)
Professional title^a^	
Senior nurse	9 (75.00%)
Associate chief nurse	3 (25.00%)
Experience providing internet-based nursing consultation	
1–3 years	5 (41.67%)
>3 years	7 (58.33%)
Number of consultations during the preceding 6 months	
<50	6 (50.00％)
50–99	2 (16.67％)
100–150	1 (8.33％)
>150	3 (25.00％)

a “Position” refers to whether the participant held a formal nursing management or other designated position. “Professional title” refers to the professional rank within the Chinese nursing professional title system.

### 3.2 Qualitative research findings

After analyzing the interview contents, nurses' experiences and perceptions of participating in internet-based nursing consultations were categorized into the following four major themes and nine sub-themes: 1. Perceived value of internet-based nursing consultation; 2. Barriers to implementation of internet-based nursing consultation; 3. Heightened risk perception and responsibility pressure; 4. Negotiating work demands and resource support. A table summarizing the themes, subthemes, and representative quotations was presented in the (Supplementary Table S2 -themes-subthemes-representative quotations).

#### 3.2.1 Theme 1: perceived value of internet-based nursing consultation

Participants perceived internet-based nursing consultation as a convenient and accessible way to provide nursing support to families with newborns while also offering nurses opportunities to enhance their professional value and sense of accomplishment. They described the service as extending nursing support beyond the temporal and spatial constraints of face-to-face care and as providing benefits for both families and nurses.

### 3.3 Highlight clinical service value

Participants perceived internet-based nursing consultation as a convenient way to address some practical challenges faced by families with newborns, particularly difficulties accessing healthcare services and obtaining timely consultations. The following were descriptions from several typical respondents:

“Newborns have weaker immune systems, and going to the hospital can be quite inconvenient for families. Internet-based nursing consultation can address parents’ urgent concerns without requiring them to travel to the hospital.” (N2)

Participants learned from parents' feedback that the service could also alleviate anxiety among the parents themselves and other caregivers.

“Many new parents often feel overwhelmed and anxious when they encounter newborn care issues. They often tell us that internet-based nursing consultations gives them professional guidance and makes them less anxious” (N3)

Participants considered that the service provided a reliable nursing support for families and perceived that it could reduce the risk of misinformation.

“Many parents now look for newborn care information on social media, but some of it is not reliable and may mislead them. Internet-based nursing consultation allows them to receive professional advice without leaving home.” (N12)

Participants noted that the service was currently offered free of charge and perceived that this could reduce the financial burden on families.

“The service is currently free, so parents do not have to pay for the consultation. I think this can reduce the financial burden on families.” **(N8)**

### 3.4 Enhance personal professional value

Participants stated that participating in internet-based nursing consultation signified high recognition from both the hospital and neonates' families of the nurses' professional competence. Moreover, participants described that it allowed nurses to continuously consolidate their professional skills and expand their knowledge base through daily practice. Participants agreed that participating in the service enhanced their professional expertise, while also provided a strong sense of professional achievement and value affirmation when helping parents and other caregivers solve practical problems.

“I feel proud to participate in the consultation service. When I help parents solve a newborn care problem and receive their thanks, I can really feel the value of my work.” (N1)

“While answering parents’ questions, I can also learn new things and strengthen my nursing skills.” (N12)

## 4 Barriers to implementation of internet-based nursing consultation

Participants reported concerns that the smooth operation of internet-based nursing consultations was constrained by multiple factors, including imperfect platform functionality, poor consultation matching, and insufficient platform promotion.

### 4.1 Imperfect platform functionality

Participants reported that the platform exhibited significant shortcomings in core functions such as triage and notification.

“There is no effective triage mechanism. After parents submit a request, it cannot be directed to the nurse with the appropriate specialty.” (N7)

“The consultation questions are not clearly categorized, so parents may not know which nurse is appropriate for their problem. This can lead to mismatched inquiries and waste time for both sides.” (N8)

“The platform does not notify us when there is a new consultation. Sometimes we miss the request, which can delay the response.” (N9)

Participants perceived the influence of display order (based on the first letter of the Pinyin spelling of the surname) on caregiver selection and consultation distribution.

“The platform currently lists nurses according to the Pinyin initial of their surnames. Those appearing earlier in the list tend to receive more consultation requests, while those later receive fewer.” (N5)

Participants described, based on parents’ feedback, that age-friendly platform design was perceived as important for improving usability.

“Some parents have reported that when elderly caregivers encounter issues while caring for newborns, they often struggle to utilize this platform.” (N4)

Furthermore, participants perceived the lack of standardization in nurses' professional profiles as contributing to inefficiencies and expressed concerns that it might increase the likelihood of case mismatches.

“The nurse profiles are too general. They should clearly show each nurse's specialty, such as wound and ostomy care or lactation.” (N10)

### 4.2 Inadequate consultation matching and information acquisition

Participants described encountering a substantial number of non-specialized questions and issues beyond the nursing scope, which they perceived as contributing to their workload.

“Many online questions are actually about medical diagnosis or treatment, which is beyond the nursing scope. Some are not even related to newborn care. These questions take a lot of time but are difficult for us to answer professionally.” (N1)

“Many parents do not look at the nurse's specialty before submitting a request. They may choose someone who is not suitable for their question, especially when the problem is unrelated to newborn care.” (N11)

Participants noted that the uneven distribution of specialist nurse resources further exacerbated the matching problem.

“There are very few specialists in some areas. For example, newborn skin problems are common, but we have only a few wound care specialists. They therefore receive a lot of pressure, and general nurses often need to consult them when we receive these questions.” (N1)

Participants described that relying on text and images for communication was limit. They perceived parental descriptions of symptoms or problems were often inaccurate or incomplete, making it difficult to assess the infant's condition and provide precise guidance.

“Internet-based nursing consultation relies mainly on text, so sometimes it is difficult to understand exactly what the parents mean. Their descriptions may be incomplete or subjective.” (N4)

“With only the parents’ descriptions and pictures, it can be difficult to assess the baby accurately. We cannot directly observe things like the baby's mental state or subtle physical signs, so sometimes we have to communicate back and forth for a long time.” (N3)

### 4.3 Lack of institutional development and insufficient platform promotion

Some participants noted that the free model could encourage frivolous or ineffective consultations.

“I think a small consultation fee could be considered. It might reduce unnecessary questions and also reflect the professional value of nurses.” (N3)

Some participants highlighted the lack of policy support and incentive mechanisms.

“We don't receive any additional performance pay for providing this service—no one has ever mentioned it.” (N6)

Some participants perceived low platform awareness as contributing to limited platform use and suggested strengthening multi-channel promotion.

“Many families still do not know that this internet-based nursing consultation service is available, so not many people use it.” (N11)

## 5 Heightened risk perception and responsibility pressure

Participants perceived that, compared with in-person care, internet-based consultations—characterized by incomplete information, the permanence of text records, and unclear liability boundaries—involved greater perceived risks related to clinical judgment, disputes, reputation, and online dissemination. They described these concerns as contributing their psychological burden and heightened sense of responsibility, and some expressed that such experiences might reduce their enthusiasm for this work.

### 5.1 Clinical judgment risks

In the online setting, nurses perceived that inaccurate assessment or guidance might delay treatment, especially given how quickly a newborn's condition could change. Participants also expressed apprehension about handling questions beyond their expertise, which they felt could lead to disputes and liability.

“I worry that incomplete information or a wrong judgment could lead to inappropriate advice and delay the baby's treatment. Newborns can deteriorate quickly, so I have to be very careful when answering.” (N8)

“I worry that incomplete assessment or inadequate guidance might negatively impact the child's treatment outcome, leading to parental dissatisfaction and potential disputes.” (N5)

“I'm concerned about not being able to give professional advice and fear that an improper response could trigger a medical dispute” (N4)

### 5.2 Communication and reputational risks

Participants noted that text-based replies were permanent and traceable. Every response could be captured in a screenshot and shared by parents. This demanded extreme precision and caution in wording. Nurses also worried that poorly chosen words might spark controversy or be maliciously disseminated, leading to negative publicity, which further compounds their stress.

“Unlike face-to-face communication, everything we say online is recorded. So, we have to be very careful with our wording and think carefully before sending a reply.” (N1)

“If a parent is unhappy with our reply, they could take a screenshot and post it online. That could affect our personal reputation and even the hospital's reputation.” (N12)

## 6 Negotiating work demands and resource support

Participants reported that work experiences varied significantly based on factors like time management, resource availability, and case assignment.

### 6.1 Flexible working hours and workload balance

Participants considered that internet-based nursing consultation offered scheduling flexibility. Several participants described using fragmented time during their working day to handle consultations, which they perceived as not increasing their overall burden.

“I usually arrange the consultation slots according to my schedule. I use fragmented time, such as breaks or evenings, to answer questions. It generally doesn’t interfere with my clinical work.” (N6)

“I usually open consultation slots during my shifts. If I come across a question, I'm not sure about, I can ask my colleagues nearby.” (N12)

However, some nurses reported that due to heavy primary workloads or uneven case distribution, they might use personal time or miss response deadlines.

“I open consultation slots during working hours, but internet-based nursing consultation is an additional task. When the clinical workload is heavy, I may not have time to check the platform, and delayed replies make me feel more stressed.” (N5)

### 6.2 Resource support and training needs

Participants agreed that organizational support from the hospital and department provided a foundational basis for the service.

“The hospital has provided some support, such as setting up a newborn nursing consultation module in the app. The Nursing Department also selects experienced nurses to provide the service.” (N6)

“When we encounter a difficult question, we can discuss it with colleagues in the department. We can support each other and then give the parents an answer.” (N12)

However, participants perceived the available training as insufficient and expressed concern that it did not adequately prepare them for complex consultations or support consistent consultation quality.

“The training provided by the hospital is not comprehensive enough. It mainly covers basic topics, such as breastfeeding and medication, but less attention is given to more complex problems, such as newborn skin problems or wound and ostomy care. When I encounter these questions, I sometimes feel that I don’t know enough to give a precise answer.” (N9)

## 7 Discussion

This qualitative study explored nurses’ experiences of providing internet-based nursing consultation for newborn caregivers. Four themes were identified: 1. Perceived value of internet-based nursing consultation; 2. Barriers to implementation of internet-based nursing consultation; 3. Heightened risk perception and responsibility pressure; 4. Negotiating work demands and resource support. Overall, the findings suggest that internet-based nursing consultation was perceived as a potentially valuable extension of specialized nursing services, particularly because participants perceived that it improved access to professional nursing guidance and provided nurses with opportunities for professional recognition and learning. However, these benefits were accompanied by challenges related to platform functionality, consultation matching, clinical uncertainty, communication risks, workload, and organizational support. Importantly, participants did not describe these experiences uniformly; the perceived benefits of flexible consultation and professional fulfillment coexisted with concerns about workload, uncertainty, and responsibility.

### 7.1 Internet-based nursing consultation provides value for families and nurses

Participants perceived internet-based nursing consultation as a convenient way to provide timely nursing support to newborn caregivers. This was particularly relevant to families caring for newborns, for whom travelling to hospital can be inconvenient and stressful. Participants also perceived that the service helped to reduce parental anxiety and, when consultations were provided without charge, reducing the immediate financial burden on families. These findings are consistent with previous studies reporting that internet-based neonatal and breastfeeding nursing consultation can improve access to nursing support and generate social and economic benefits (8, 13, 14).

The findings also extend previous work by highlighting the perceived professional value of consultation for nurses themselves. Participants described feelings of accomplishment when their advice helped families solve newborn-care problems, while the need to address unfamiliar questions encouraged nurses to review and consolidate their professional knowledge. This finding is particularly relevant because participation in the service in the present setting was restricted to experienced nurses with specialist qualifications. Thus, internet-based nursing consultation may provide an additional setting in which specialist nurses can apply and demonstrate their expertise rather than simply representing an additional communication channel. Professional accomplishment has been associated with nurses' professional development and workforce retention, suggesting that opportunities to use specialized knowledge may have value for both individual nurses and organizations (15).

At the same time, these findings should not be interpreted as demonstrating that internet-based consultation improves clinical outcomes. Participants described perceived benefits in accessibility, parental support, and professional fulfillment, but these outcomes were not quantitatively evaluated in this study. Future research should therefore assess service effectiveness from multiple perspectives, including parents and other caregivers, nurses, and healthcare organizations, using outcomes such as satisfaction, appropriateness of referral, consultation resolution, and subsequent healthcare utilization.

### 7.2 Platform functionality and consultation matching remain important implementation barriers

Although participants recognized the value of internet-based consultation, they identified several platform-level barriers that could limit its effectiveness. These included inadequate triage and notification functions, difficulty identifying nurses according to their specialist expertise, uneven distribution of consultation requests, and usability problems for some caregivers. These findings suggest that the effectiveness of an online nursing service depends not only on nurses' professional competence but also on whether the digital infrastructure appropriately connects the right caregiver with the right professional (16).

The consultation-matching issue was particularly important in the neonatal context. Participants described situations in which caregivers submitted questions outside the responding nurse's specialty, requiring nurses to seek assistance from colleagues. This problem may be more pronounced for specialized neonatal services because neonatal nursing encompasses multiple areas of expertise, while the number of nurses with particular specialist qualifications may be limited. Rather than interpreting this finding simply as an individual competency problem, it may therefore be more appropriate to regard consultation matching as an organizational and platform-design issue.

Participants' descriptions support the potential value of more structured presentation of nurses' specialist expertise and more appropriate allocation of consultation requests. However, such approaches should initially be understood as service-design considerations rather than established solutions. Evidence on telehealth implementation suggests that accessibility, appropriateness of care, usability, and system-level factors all need to be considered when evaluating digital healthcare models (17).

The reported difficulty experienced by some older caregivers also highlights the importance of digital inclusion. In neonatal care, the primary caregiver may change over time, and grandparents may become involved in infant care. Therefore, designing a service only for digitally proficient younger users may inadvertently create a new access barrier. Recent evidence indicates that digital exclusion among older adults is associated with socioeconomic, health-related, and contextual disadvantages and that age-friendly design, digital skills support, affordable access, and non-digital alternatives may be necessary to reduce inequity (18). Accordingly, future development of neonatal internet-based nursing consultation should consider not only technical functionality but also equitable access, usability, and alternative pathways for caregivers who cannot effectively use digital platforms.

Moreover, if compensation were directly linked to the number of consultations, it could potentially create incentives to prioritize consultation volume rather than quality. Evidence concerning pay-for-performance in healthcare suggests that financial incentives may produce mixed effects and can have unintended consequences depending on how they are designed (19). Therefore, the present findings support further evaluation of sustainable compensation and institutional support mechanisms, rather than recommending a specific fee-based model. Future research should incorporate the perspectives of nurses, parents and other caregivers, administrators, and policymakers and consider affordability, workload, service quality, equity, and sustainability when evaluating different models.

### 7.3 Remote consultation creates a distinctive patient-safety and professional responsibility context

A central finding of this study was that nurses experienced heightened responsibility when providing advice remotely. Participants were concerned that incomplete information, poor-quality images, or questions beyond their expertise could make it difficult to assess an infant accurately. These concerns are particularly relevant in neonatal care because infants may deteriorate rapidly and because remote consultation cannot fully reproduce the information available during face-to-face assessment. Importantly, these findings concern nursing consultation and assessment rather than online diagnosis. The purpose of internet-based nursing consultation should therefore be understood as providing nursing guidance within an appropriate professional scope, identifying situations requiring further assessment, and directing caregivers to appropriate in-person services when remote information is insufficient. This distinction is important for patient safety and professional accountability.

The participants' concerns are consistent with broader evidence that remote consultations may alter communication, information exchange, care coordination, and patient engagement, creating specific patient-safety risks (20). More recent evidence has similarly emphasized that patient safety in virtual consultations should be evaluated through provider-, patient-, and system-related factors as well as tangible outcomes such as adverse events and healthcare utilization (21).

Therefore, the findings support the need for clear service boundaries, structured information collection, appropriate escalation pathways, and professional training, rather than the use of internet-based nursing consultation as a substitute for direct clinical assessment. When nurses cannot obtain sufficient information or identify a problem within their scope of practice, referral for face-to-face assessment should remain an important safety mechanism.

Participants also expressed concerns about the permanence of online communication and the possibility that screenshots could be shared outside the original consultation context. These concerns suggest that communication quality and privacy protection are important components of service governance. However, the present qualitative data do not allow conclusions about specific legal liabilities or the effectiveness of particular technical safeguards.

### 7.4 Professional support and workload determine whether flexibility becomes a benefit or a burden

The findings showed that flexible scheduling was experienced differently by participants. Some nurses, such as N6, were able to use fragmented periods of time to complete consultations without substantial interference with their routine clinical work. For nurses with less consultation experience, opening consultation slots during working hours could also provide immediate access to colleagues when difficult questions arose. These findings suggest that flexibility may facilitate integration of internet-based nursing consultation into existing workflows. However, flexibility did not necessarily mean reduced workload. N5 described situations in which heavy clinical workloads made it difficult to respond promptly to internet-based nursing consultations, turning the service into an additional source of stress. Thus, the effect of internet-based nursing consultation on workload appeared to depend not simply on the availability of flexible working hours but also on clinical workload, consultation volume, case complexity, and access to collegial support (16). This finding is important because it prevents the service from being conceptualized as a cost-free extension of nurses' existing work. Organizational support, workload recognition, protected time, and access to specialist colleagues may be necessary if hospitals expect nurses to provide sustained internet-based nursing consultation services. The present findings support strengthening institutional support and training, particularly in areas where consultation demand is high but specialist resources are limited.

Participants also reported that existing training did not sufficiently cover complex neonatal questions. This suggests that training for internet-based nursing consultation should extend beyond general communication skills and basic neonatal care to include remote information collection, recognition of situations unsuitable for online management, communication of uncertainty, escalation and referral, and management of questions outside one's specialty.

## 8 Strengths and limitations

This study has several strengths. First, the study was conducted within an established real-world internet-based nursing consultation service, allowing the findings to reflect nurses' actual experiences and challenges in delivering internet-based nursing consultation alongside their routine clinical responsibilities. In addition, by focusing on the perspectives of nurses as service providers, this study complements the existing literature, which has predominantly employed quantitative designs and examined the effects of internet-based nursing services from the perspectives of patients and families. The qualitative approach further enabled an in-depth exploration of the complex experiences, challenges, and needs that may not be adequately captured by quantitative measures.

This study also has several limitations. First, all participants were recruited from a single NICU and met the institutional eligibility requirements for providing internet-based nursing consultation. Although these characteristics were appropriate to the study context, the relatively homogeneous sample and single-center setting may limit the transferability of the findings to nurses with different professional backgrounds and to hospitals with different eligibility criteria or service models. Second, the interviewer worked in the same NICU as the participants. Although the interviewer had no managerial authority, this relationship may have influenced participants' responses. Moreover, selected quotations were translated from Mandarin into English; although bilingual researchers checked the translations against the original transcripts, subtle differences in meaning may have occurred during translation.

## 9 Conclusion

This study explored NICU nurses' experiences of providing internet-based nursing consultation and identified perceived benefits, barriers, and support needs associated with this service. The findings suggest that internet-based nursing consultation may provide a convenient channel for delivering professional nursing guidance to parents and other caregivers and may also present challenges related to workload, professional responsibility, risk management, and organizational support.

## 10 Implications for practice and future research

The findings suggest that the sustainable development of internet-based nursing consultation may require coordination across three levels. At the service level, consultation pathways should clearly define the scope of nursing consultation, establish mechanisms for identifying cases that require face-to-face assessment, and provide training for managing uncertainty and complex questions. At the organizational level, hospitals should consider workload, specialist resource distribution, protected time, collegial support, and appropriate professional recognition. At the platform level, future development should prioritize usability, timely notifications, appropriate consultation matching, and equitable access rather than simply adding technological complexity.

Future research should also move beyond nurses' perspectives. Parents and other caregivers should be included to evaluate accessibility, affordability, satisfaction, trust, and perceived usefulness. Administrators and policymakers should be involved in examining organizational sustainability and governance. Quantitative studies could subsequently evaluate consultation resolution rates, referral rates, response times, caregiver satisfaction, workload, and safety outcomes. Such evidence would help determine which aspects of internet-based nursing consultation genuinely improve care and which may unintentionally introduce new barriers or risks.

## Data Availability

The datasets presented in this article are not readily available because the dataset contains qualitative interview data from NICU nurses and includes potentially identifiable information. Therefore, the dataset is not publicly available due to ethical and privacy restrictions. Requests to access the datasets should be directed to Jiahua Zhang zjh13784310227@163.com
