# Supplementary material for: NICU nurses’ experiences of providing internet-based nursing consultation services: a descriptive qualitative study
Source: Front Pediatr. 2026 Oct 8;14:1950395. doi: 10.3389/fped.2026.1950395 (PMC13687276; doi:10.3389/fped.2026.1950395)
Supplement: Supplementary file 1 [file Table1.docx]

**Table 1. The coding framework**

| **Participant** | **Meaning unit** | **Condensed**  **meaning unit** | **Code** | **Subtheme** | **Theme** |
| --- | --- | --- | --- | --- | --- |
| **N2** | Newborns have weaker immune systems, and going to the hospital can be quite inconvenient for families. Internet-based nursing consultation can address parents’ urgent concerns without requiring them to travel to the hospital. | Newborns are vulnerable and hospital visits are inconvenient; online nursing consultation can address urgent parental concerns remotely. | Convenient access to nursing support | **1.1 Highlight clinical service value** | **Theme 1. Perceived value of internet-based nursing consultation** |
| **N3** | Many new parents often feel overwhelmed and anxious when they encounter newborn care issues. They often tell us that internet-based nursing consultations gives them professional guidance and makes them less anxious | New parents feel anxious about newborn care; professional online guidance can make them feel less anxious. | Alleviating parental anxiety | **1.1 Highlight clinical service value** | **Theme 1. Perceived value of internet-based nursing consultation** |
| **N8** | Many parents now look for newborn care information on social media, but some of it is not reliable and may mislead them. Internet-based nursing consultation allows them to receive professional advice without leaving home. | Social-media information may be unreliable; online nursing consultation provides professional advice at home. | Reduced financial burden | **1.1 Highlight clinical service value** | **Theme 1. Perceived value of internet-based nursing consultation** |
| **N1** | The service is currently free, so parents do not have to pay for the consultation. I think this can reduce the financial burden on families. | Free consultation means parents do not pay and is perceived as reducing families’ financial burden. | Professional recognition and accomplishment | **1.2 Enhance personal professional value** | **Theme 1. Perceived value of internet-based nursing consultation** |
| **N12** | I feel proud to participate in the consultation service. When I help parents solve a newborn care problem and receive their thanks, I can really feel the value of my work. | Helping parents solve newborn-care problems and receiving thanks creates pride and a sense of work value. | Professional knowledge development | **1.2 Enhance personal professional value** | **Theme 1. Perceived value of internet-based nursing consultation** |
| **N7** | While answering parents’ questions, I can also learn new things and strengthen my nursing skills. | Answering parents’ questions provides opportunities to learn and strengthen nursing skills. | Inadequate consultation triage | **2.1 Imperfect platform functionality** | **Theme 2. Barriers to implementation of internet-based nursing consultation** |
| **N9** | There is no effective triage mechanism. After parents submit a request, it cannot be directed to the nurse with the appropriate specialty. | The platform lacks effective triage and cannot direct requests to nurses with the appropriate specialty. | Inadequate notification | **2.1 Imperfect platform functionality** | **Theme 2. Barriers to implementation of internet-based nursing consultation** |
| **N5** | The consultation questions are not clearly categorized, so parents may not know which nurse is appropriate for their problem. This can lead to mismatched inquiries and waste time for both sides. | Unclear question categories make it difficult for parents to select an appropriate nurse, leading to mismatches and wasted time. | Uneven case allocation | **2.1 Imperfect platform functionality** | **Theme 2. Barriers to implementation of internet-based nursing consultation** |
| **N4** | The platform does not notify us when there is a new consultation. Sometimes we miss the request, which can delay the response. | Lack of new-consultation notifications can cause missed requests and delayed responses. | Age-related platform usability barriers | **2.1 Imperfect platform functionality** | **Theme 2. Barriers to implementation of internet-based nursing consultation** |
| **N10** | The platform currently lists nurses according to the Pinyin initial of their surnames. Those appearing earlier in the list tend to receive more consultation requests, while those later receive fewer. | Nurse display order by surname Pinyin initial is perceived as producing unequal consultation volumes. | Inadequate display of nurse specialties | **2.1 Imperfect platform functionality** | **Theme 2. Barriers to implementation of internet-based nursing consultation** |
| **N1** | Some parents have reported that when elderly caregivers encounter issues while caring for newborns, they often struggle to utilize this platform. | Elderly caregivers may struggle to use the consultation platform. | Out-of-scope consultation requests | **2.2 Inadequate consultation matching and information acquisition** | **Theme 2. Barriers to implementation of internet-based nursing consultation** |
| **N11** | The nurse profiles are too general. They should clearly show each nurse’s specialty, such as wound and ostomy care or lactation. | Nurse profiles are overly general and should clearly display specialty expertise. | Limited awareness of nurse specialization | **2.2 Inadequate consultation matching and information acquisition** | **Theme 2. Barriers to implementation of internet-based nursing consultation** |
| **N1** | Many online questions are actually about medical diagnosis or treatment, which is beyond the nursing scope. Some are not even related to newborn care. These questions take a lot of time but are difficult for us to answer professionally. | Many questions concern diagnosis/treatment or non-newborn issues, fall outside nursing scope, and consume time while being difficult to answer professionally. | Uneven specialist resource distribution | **2.2 Inadequate consultation matching and information acquisition** | **Theme 2. Barriers to implementation of internet-based nursing consultation** |
| **N3** | Many parents do not look at the nurse’s specialty before submitting a request. They may choose someone who is not suitable for their question, especially when the problem is unrelated to newborn care. | Parents may ignore nurses’ specialties and select a nurse who is not appropriate for their question. | Incomplete information for clinical assessment | **2.2 Inadequate consultation matching and information acquisition** | **Theme 2. Barriers to implementation of internet-based nursing consultation** |
| **N3** | There are very few specialists in some areas. For example, newborn skin problems are common, but we have only a few wound care specialists. They therefore receive a lot of pressure, and general nurses often need to consult them when we receive these questions. | Some specialties have too few nurses, creating pressure on specialists and requiring general nurses to seek their help. | Potential value of a charging mechanism | **2.3 Lack of institutional development and insufficient platform promotion** | **Theme 2. Barriers to implementation of internet-based nursing consultation** |
| **N6** | Internet-based nursing consultation relies mainly on text, so sometimes it is difficult to understand exactly what the parents mean. Their descriptions may be incomplete or subjective. | Text-based consultation makes parents’ meanings difficult to understand because descriptions may be incomplete or subjective. | Insufficient incentive mechanisms | **2.3 Lack of institutional development and insufficient platform promotion** | **Theme 2. Barriers to implementation of internet-based nursing consultation** |
| **N11** | With only the parents’ descriptions and pictures, it can be difficult to assess the baby accurately. We cannot directly observe things like the baby’s mental state or subtle physical signs, so sometimes we have to communicate back and forth for a long time. | Parent descriptions and pictures are insufficient for direct observation, making accurate assessment difficult and requiring prolonged back-and-forth communication. | Low service awareness | **2.3 Lack of institutional development and insufficient platform promotion** | **Theme 2. Barriers to implementation of internet-based nursing consultation** |
| **N8** | I think a small consultation fee could be considered. It might reduce unnecessary questions and also reflect the professional value of nurses. | A small fee is perceived as potentially reducing unnecessary questions and reflecting nurses’ professional value. | Risk of inaccurate clinical judgment | **3.1 Clinical judgment risks** | **Theme 3. Heightened risk perception and responsibility pressure** |
| **N5** | We don't receive any additional performance pay for providing this service—no one has ever mentioned it. | No additional performance pay is provided for consultation work. | Fear of adverse consequences and disputes | **3.1 Clinical judgment risks** | **Theme 3. Heightened risk perception and responsibility pressure** |
| **N4** | Many families still do not know that this internet-based nursing consultation service is available, so not many people use it. | Many families are unaware of the service, which is perceived as contributing to low use. | Uncertainty about professional adequacy | **3.1 Clinical judgment risks** | **Theme 3. Heightened risk perception and responsibility pressure** |
| **N1** | I worry that incomplete information or a wrong judgment could lead to inappropriate advice and delay the baby’s treatment. Newborns can deteriorate quickly, so I have to be very careful when answering. | Incomplete information or incorrect judgment may lead to inappropriate advice and delayed treatment, requiring great caution because newborns can deteriorate quickly. | Permanence and traceability of communication | **3.2 Communication and reputational risks** | **Theme 3. Heightened risk perception and responsibility pressure** |
| **N12** | I worry that incomplete assessment or inadequate guidance might negatively impact the child’s treatment outcome, leading to parental dissatisfaction and potential disputes. | Incomplete assessment or inadequate guidance is perceived as potentially affecting treatment outcomes and causing dissatisfaction or disputes. | Risk of screenshot dissemination and reputational damage | **3.2 Communication and reputational risks** | **Theme 3. Heightened risk perception and responsibility pressure** |
| **N6** | I’m concerned about not being able to give professional advice and fear that an improper response could trigger a medical dispute | Inability to provide professional advice or an improper response may trigger a medical dispute. | Flexible use of fragmented time | **4.1 Flexible working hours and workload balance** | **Theme 4. Negotiating work demands and resource support** |
| **N12** | Unlike face-to-face communication, everything we say online is recorded. So, we have to be very careful with our wording and think carefully before sending a reply. | Online communication is permanently recorded, requiring careful wording and deliberation before replying. | Using collegial support to manage uncertainty | **4.1 Flexible working hours and workload balance** | **Theme 4. Negotiating work demands and resource support** |
| **N5** | If a parent is unhappy with our reply, they could take a screenshot and post it online. That could affect our personal reputation and even the hospital’s reputation. | Parents can screenshot and post replies online, potentially affecting nurses’ and the hospital’s reputations. | Workload-related consultation stress | **4.1 Flexible working hours and workload balance** | **Theme 4. Negotiating work demands and resource support** |
| **N6** | I usually arrange the consultation slots according to my schedule. I use fragmented time, such as breaks or evenings, to answer questions. It generally doesn’t interfere with my clinical work. | Consultation slots can be arranged around the nurse’s schedule and handled during fragmented time without generally interfering with clinical work. | Institutional organizational support | **4.2 Resource support and training needs** | **Theme 4. Negotiating work demands and resource support** |
| **N12** | I usually open consultation slots during my shifts. If I come across a question, I’m not sure about, I can ask my colleagues nearby. | Opening consultation slots during shifts allows nurses to seek nearby colleagues’ help with uncertain questions. | Departmental collegial support | **4.2 Resource support and training needs** | **Theme 4. Negotiating work demands and resource support** |
| **N9** | I open consultation slots during working hours, but internet-based nursing consultation is an additional task. When the clinical workload is heavy, I may not have time to check the platform, and delayed replies make me feel more stressed. | Online consultation is an additional task; heavy clinical workload can prevent timely platform checks and delayed replies increase stress. | Insufficient professional training | **4.2 Resource support and training needs** | **Theme 4. Negotiating work demands and resource support** |

Note: Meaning units and condensed meaning units were derived from participants’ accounts and therefore represent participants’ perceptions, experiences, views, and concerns rather than objectively measured effects or independently verified causal relationships.
