# Supplementary material for: NICU nurses’ experiences of providing internet-based nursing consultation services: a descriptive qualitative study
Source: Front Pediatr. 2026 Oct 8;14:1950395. doi: 10.3389/fped.2026.1950395 (PMC13687276; doi:10.3389/fped.2026.1950395)
Supplement: Supplementary file 2 [file Table2.docx]

**Table 2.** Summary of the themes, subthemes, and representative quotations

| **Theme** | **Subtheme** | **Concise description** | **Representative quotation** |
| --- | --- | --- | --- |
| **1. Perceived value of internet-based nursing consultation** | **1.1 Highlight clinical service value** | Online consultation was perceived as a convenient and accessible way to provide timely nursing support, reduce barriers to hospital visits, alleviate parental anxiety, and reduce families’ financial burden. | “Newborns have weaker immune systems, and going to the hospital can be quite inconvenient for families. Online consultation can address parents’ urgent concerns without requiring them to travel to the hospital.” (N2) |
|  | **1.2 Enhance personal professional value** | Participants described online consultation as providing professional recognition and a sense of accomplishment, as well as opportunities to strengthen nursing knowledge and skills through practice. | “I feel proud to participate in the consultation service. When I help parents solve a newborn care problem and receive their thanks, I can really feel the value of my work.” (N1) |
| **2. Barriers to implementation of internet-based nursing consultation** | **2.1 Imperfect platform functionality** | Participants perceived limitations in triage, notifications, case allocation, platform usability, and participants expressed concern that these limitations could reduce consultation efficiency and contribute to uneven workload distribution. | “There is no effective triage mechanism. After parents submit a request, it cannot be directed to the nurse with the appropriate specialty.” (N7) |
|  | **2.2 Inadequate consultation matching and information acquisition** | Participants described mismatches between parents’ questions and nurses’ specialties, limited specialist resources, and incomplete information obtained through text and images as making accurate and efficient guidance more difficult. | “There are very few specialists in some areas. For example, newborn skin problems are common, but we have only a few wound care specialists. They therefore receive a lot of pressure, and general nurses often need to consult them when we receive these questions.” (N1) |
|  | **2.3 Lack of institutional development and insufficient platform promotion** | Participants perceived limited policy and incentive support, uncertainty about the free-service model, and low public awareness as constraining the further development and uptake of the service. | “I think a small consultation fee could be considered. It might reduce unnecessary questions and also reflect the professional value of nurses.” (N3) |
| **3. Heightened risk perception and responsibility pressure** | **3.1 Clinical judgment risks** | Participants expressed concerns that incomplete information, inability to directly assess infants, and questions beyond nurses’ expertise could contribute to inappropriate advice, delayed treatment, complaints, and disputes. | “I worry that incomplete information or a wrong judgment could lead to inappropriate advice and delay the baby’s treatment. Newborns can deteriorate quickly, so I have to be very careful when answering.” (N8) |
|  | **3.2 Communication and reputational risks** | Participants described the permanence and traceability of online communication as making them more cautious about wording, while the possibility of screenshots being shared online was perceived as creating concerns about personal and institutional reputation. | “Unlike face-to-face communication, everything we say online is recorded. So we have to be very careful with our wording and think carefully before sending a reply.” (N1) |
| **4. Negotiating work demands and resource support** | **4.1 Flexible working hours and workload balance** | Participants described flexible scheduling as allowing consultations to be integrated into fragmented periods of time, while heavy clinical workloads were perceived as making online consultation an additional source of stress. | “I usually arrange the consultation slots according to my schedule. I use fragmented time, such as breaks or evenings, to answer questions. It generally doesn’t interfere with my clinical work.” (N6) |
|  | **4.2 Resource support and training needs** | Participants described hospital and departmental support, including organizational arrangements and collegial assistance, as facilitating consultation delivery, while existing professional training was considered insufficient for complex consultation needs. | “The training provided by the hospital is not comprehensive enough. It mainly covers basic topics, such as breastfeeding and medication, but less attention is given to more complex problems, such as newborn skin problems or wound and ostomy care.” (N9) |

**Notes**: The four major themes are now explained as follows:

1.	Perceived value of internet-based nursing consultation

This theme captures nurses’ perceptions of the benefits and meaningfulness of providing internet-based nursing consultation. Its sub-themes describe the value perceived for newborn caregivers, including improved access to professional support, as well as the professional value experienced by nurses, such as professional accomplishment and opportunities to consolidate knowledge.

2.	Barriers to implementation of internet-based nursing consultation

This theme reflects difficulties arising from the service and platform environment that may limit the effective delivery of online consultation. Its sub-themes address issues such as platform functionality, consultation matching, uneven distribution of consultation requests, and digital accessibility.

3.	Heightened risk perception and responsibility pressure

This theme describes nurses’ concerns about providing nursing advice when clinical information is incomplete or difficult to verify remotely. Its sub-themes capture uncertainty in remote assessment, questions outside nurses’ expertise, communication risks, and the perceived responsibility associated with online advice.

4.	Negotiating work demands and resource support

This theme reflects how nurses attempted to integrate internet-based consultation with their existing clinical responsibilities and available organizational resources. Its sub-themes address flexibility and workload, access to collegial support, specialist resources, training needs, and organizational recognition/support.
